# Spermatorrhœa

**Published:** 1884-05-20

**Authors:** J. S. Geigley

**Affiliations:** Lewiston, Ill.


### SPERMATORRHŒA.

BY J. S. GEIGLEY, M.D., LEWISTON, ILL.

There are, perhaps, few physicians who have not been called upon to treat one or more cases of the above affection, and those that have treated the greatest number will, I am sure, agree with me when I say there are few diseases the treatment of which is less satisfactory than this functional disorder of the male generative organs; for, on looking through works in which this subject is treated, we find almost numberless remedies recommended, any of which might cure, or all of which may fail. Indeed, it has almost become a maxim in medicine that the more numerous the medicines the more incurable the disease. The disease, in itself, is not serious, but the effect it has on the patient's mind is sometimes very serious. This is usually by his consulting some ignorant person, or reading some one of the innumerable advertising "treatises" that are scattered broadcast by enterprising quacks, who trade on the ignorance or misfortunes of the people.

The term "spermatorrhœa" is frequently used indiscriminately in speaking of several different conditions, thus: prostatorrhœa, nocturnal emissions, premature emissions (seminal weakness), and involuntary emissions, are commonly spoken of as spermatorrhœa, when, in reality, the term should be applied to the latter condition only. True, the difference in these conditions is more one of degree than of form; but sometimes it is a great comfort to a patient to be told that he has something other than spermatorrhœa. Not unfrequently a young man will become alarmed about a discharge that takes place after passing his urine; this is sometimes quite abundant; it also resembles seminal fluid somewhat, and if this patient has an occasional nocturnal emission, he is sure he has spermatorrhœa. This is what authors call prostatorrhœa, and is most likely caused by constipation, or possibly an old clap. While nocturnal emissions might be considered the first stage of spermatorrhœa, I am convinced that almost any healthy individual, who does not indulge in sexual intercourse, will have nocturnal emissions as often, perhaps, as every two or three weeks. They will always occur during a lascivious dream, and will not injure his health in the least; but if these occur oftener than every ten days, we must look for a cause other than celibacy.

Sometimes the patient will complain of the organism taking place at the beginning of the sexual act; or, that he has emissions when in the society of the opposite sex. This is likely to be accompanied by nocturnal emissions with dreams, and is the condition usually called seminal weakness, or premature emissions.

In true spermatorrhœa the patient will tell you he has an emission every—or nearly every—night, generally unaccompanied by dreams of an amorous nature, and is only aware that he had an emission by the appearance of his linen in the morning. He will also tell you he has lost the desire for sexual intercourse, but that a lewd picture, or the contact of a woman's dress, will sometimes cause a feeble erection, with an emission; also, at times, the least stimulation applied to the glans will act the same way; and, further, that sometimes an involnntary emission will take place independent of any cause. These patients sometimes brood over their misfortunes until they are really objects of pity, and their health begins to be seriously affected; not, perhaps, through any systemic or physical drain, but through the effect produced upon the nervous system by the loss of nervous force at each emission.

The causes of these troubles are various, but that dilated upon by most writers is masturbation. While admitting this to be a fruitful cause of the trouble, I fail to see how it should produce results differing from normal coitus. Sir James Paget says: "The mischief is due to the quantity, not to the method of the excesses." We will notice that the majority of these patients come from the middle and upper walks of life; their habits are sedentary, being either students or their employment is of such a nature as to require little physical exertion. Many of these patients are the victims of obstinate constipation, and we can easily see how a hardened mass of fæces in the rectum could produce irritability of the urethra and prostate gland by constant pressure of these organs.

As I did not commence this paper with the expectation of adding anything to our knowledge of the nature of the disease, I will report a few cases and close this already too lengthy article.

Case 1, Sept. 20th, 1882.—W., aged 17, good growth, but delicate, shows heavy dark circles under the eyes, attends school, is studious and intelligent; has been troubled with nocturnal emissions for over a year; have been increasing in frequency from the start; at present, has one almost every night; has had as many as two in one night, generally accompanied by a dream; says he has not practiced masturbation for over a year, never to excess; no sexual intercourse for over a year; had his passions frequently aroused, but not gratified, by a servant girl in the family; no involuntary emissions, but a stringy discharge after passing urine; bowels very costive, sometimes no movement for three or four days. My prescription was a pill of podophilin and hydrastin, with a globule of atropia each night, saline laxative before breakfast, a tonic mixture through the day, and a half drachm of bromide of potassium with eight drops fl. ex. gelsem. in half glass of water after eating supper. This and similar treatment was kept up about six weeks, with no further result than an improvement in the condition of the bowels. Finally, the patient becoming discouraged, I concluded to cauterize the prostatic portion of the urethra, as is recommended by some authors. I devised an instrument for carrying tinct. iodine to the part. My instrument was this: An ordinary gum catheter, No. 9, with the eyed end cut off so that the stylet would protrude about a quarter inch; now a small piece of sponge is secured over the end of the stylet with a fine silk thread, the ends of which should be left long and brought up through the catheter and secured to the ring in the other end of the stylet; this will prevent the sponge from being left in the urethra, if it should slip; the sponge should be trimmed smooth and round, and should exceed the diameter of the catheter somewhat when distended with fluid. The sponge is to be dipped in strong tinct. iodine and carefully drawn into the catheter; an ordinary gelatine capsule, softened by holding in the mouth a moment, is slipped over the end of the catheter, which is now introduced into the urethra as far as the prostatic portion, when the stylet is pushed forward and the whole instrument withdrawn. This applies the iodine to the prostatic urethra pretty thoroughly.

This instrument I used on my patient with the result of quite an inflammation of the deep urethra, and considerable pain on passing urine, which was controlled with large doses of potassium bromide. I repeated the operation on the 8th and 20th days, during which time the patient had no emissions. The amount of disturbance following the last application of iodine was very slight. From this time on the patient had no more than one or two emissions a month, and considered himself well. Outside of the local applications, the rest of the treatment was purely symptomatic.

Case 2, Dec. 7th, 1882.—R., clerk, aged 20, had been troubled with nocturnal emissions 18 months; had gradually increased in frequency; was now having from three to five a week; sometimes had a dream, sometimes not; attempted to have intercourse lately, but failed on account of the emission taking place prematurely; felt very much frightened, and concluded to consult a doctor; is feeling very badly; has severe pains in the back, pains in the head and vertigo; thinks he is going to die; said he had not practiced masturbation for some time, would not say for how long; has no appetite. Prescribed a saline laxative and a bitter tonic, and made the local application of iodine, By the second day the urethral disturbance was so great that it drew his, as well as my own attention away from the original trouble; this disturbance was controlled, however, by hot fomentations to the perineum, large doses of the bromides, with a rectal suppository of belladonna and hyoscyamus. Being a little timid about using iodine again in his case, I concluded to try electricity by means of an intro-urethral electrode (this is suggested by several writers). I made my electrode by taking a piece of three-sixteenths inch round iron, and filing all but about two and one-half inches at one end down to the size of one-eighth inch or less, The end left full size is to be the beak of the instrument, and should have a curve corresponding to the quadrant of a circle, the size of which would be about four inches; a piece of thin rubber tubing should be stretched over the small part of the instrument, until it fits snugly against the shoulder of the beak, when we have a good urethral electrode, nicely insulated at all points, except where we want the current to take effect. I attached this instrument to the negative pole of a Faradic battery, and introduced it down to the prostatic urethra, allowing it to remain about ten minutes, meanwhile passing the sponge of the positive pole from the lumbar region down to the perineum; this produced some vesical irritability for a few days. I gave this patient one sitting a week for six weeks, at the end of which time he considered himself well. In this case I gave the credit of the cure to the application of the iodine, for the condition of his penis occupied the patient's attention, to the exclusion of everything else, for about eight days, and he went three weeks without having an emission.

Case 3, Feb. 10th, 1883.—A., aged 22, farmer, has nocturnal emissions, one to three a week; has been in same condition for some time; during the last few weeks has a profuse discharge, which he thinks is seminal matter. Patient expects to get married soon, and his condition troubles him very much; had gonorrhœa several years ago; appears well nourished and in good health, but is troubled part of the time with obstinate constipation. Prescribed the usual remedies for this. Applied the prostatic electrode, but had some difficulty in passing a stricture situated well back in the membranous portion; considerable irritability back of stricture. I gave the patient a sitting a week for a month, also using the bromides and salines during this time; had but one emission, but still had some discharge. I applied the iodine to the prostatic and membranous portion thoroughly, and passed a good sized sound every other day. The discharge ceased in a few days.

I could report several more cases of the same kind, but it would be useless, as they are so near alike. In conclusion will say, I think the important part of the treatment is the breaking up of the *habit* of these emissions; this being done, it encourages the patient and gives him the confidence necessary for an intelligent co-operation in the treatment of the case. In my opinion, the local treatment adopted in these cases broke the habit by the shock which it produeed in the neighborhood of the openings of the ejaculatory ducts; also, the effect produced upon the *mind* of the patient by the use of apparatus (which, as he thinks, reaches the diseased part) must not be overlooked.—*Peoria Medical Monthly.*

